# HLA-DQ and HLA-DRB1 alleles associated with Henoch-Schönlein purpura nephritis in Finnish pediatric population: a genome-wide association study

**DOI:** 10.1007/s00467-021-04955-7

**Published:** 2021-02-16

**Authors:** Mikael Koskela, Julia Nihtilä, Elisa Ylinen, Kaija-Leena Kolho, Matti Nuutinen, Jarmo Ritari, Timo Jahnukainen

**Affiliations:** 1 Children’s Hospital, Pediatric Research Center, University of Helsinki, Helsinki University Hospital, Helsinki, Finland; 2 Department of Pediatric Nephrology and Transplantation, New Children’s Hospital, University of Helsinki and Helsinki University Hospital, PO Box 347, Stenbäckinkatu 9, 00029 HUS, Helsinki, Finland; 3 University of Helsinki, Helsinki, Finland; 4 Finnish Red Cross Blood Service, Helsinki, Finland; 5 Faculty of Medicine and Health Technology, Tampere University, Tampere, Finland; 6 Department of Children and Adolescents, Oulu University Hospital, Oulu, Finland; 7 PEDEGO Research Unit, Research Unit for Pediatrics, Dermatology, Clinical Genetics, Obstetrics and Gynecology, Medical Research Center Oulu (MRC Oulu), Oulu, Finland

**Keywords:** Crohn’s disease, Inflammatory bowel disease, Children, Genetics, IgA vasculitis

## Abstract

**Background:**

The pathophysiology of Henoch-Schönlein purpura (HSP) is still unclear, but several findings suggest that genetic factors may influence disease susceptibility. We aimed to perform a genome-wide association study (GWAS) in pediatric HSP patients with an emphasis on severe HSP nephritis.

**Methods:**

The study included 46 HSP patients, 42 of whom had undergone kidney biopsy. Forty-nine pediatric patients with an inflammatory bowel disease (IBD) served as an autoimmune disease control group while Finnish bone marrow and blood donors represented the general reference population (*n* = 18,757). GWAS was performed for HSP and IBD samples in a case-control manner against the reference population. The analysis also included imputation of human leukocyte antigen (HLA) alleles.

**Results:**

GWAS analysis in HSP revealed several polymorphisms from the HLA region that surpassed the genome-wide significance level. Three HLA class II alleles were also significantly more frequent in HSP than in the reference population: DQA1*01:01, DQB1*05:01, and DRB1*01:01. Haplotype DQA1*01:01/DQB1*05:01/DRB1*01:01 occurred in 43.5% of HSP patients, whereas its frequency was 8.2% in IBD patients and 15.0% in the reference population. HSP patients with this haplotype showed similar baseline clinical findings and outcome as HSP patients negative for the haplotype. In IBD patients, no polymorphism or HLA allele appeared significant at the genome-wide level.

**Conclusions:**

Our results suggest that haplotype DQA1*01:01/DQB1*05:01/DRB1*01:01 is associated with susceptibility to HSP, but not with the severity of the kidney involvement. These HLA associations did not occur in IBD patients, suggesting that they are specific to HSP and not related to susceptibility to autoimmune diseases in general.

**Graphical abstract:**

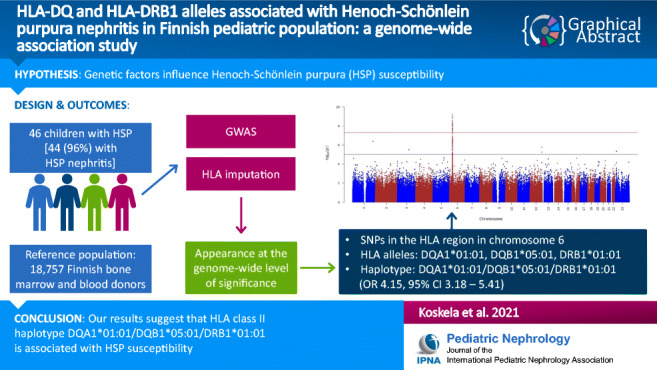

**Supplementary Information:**

The online version contains supplementary material available at 10.1007/s00467-021-04955-7.

## Introduction

Henoch-Schönlein purpura (HSP) is an IgA-mediated vasculitis occurring predominantly in childhood [1]. Purpuric rash occurs in all HSP patients, other frequently affected sites being joints, the gastrointestinal tract, and kidneys [2, 3]. Even though especially gastrointestinal involvement may rarely cause acute complications [4], the long-term outcome is principally dependent on the severity of the kidney component. Chronic kidney disease (CKD) occurs in roughly one-fifth of the patients with initial nephrotic or nephritic syndrome, whereas in patients with less severe kidney involvement, the risk of CKD is approximately 2% [5].

The occurrence of HSP has a clear seasonal variation, and up to two-thirds of HSP patients have a preceding infection; these findings point to an environmental influence in predisposition to the disease [1, 6]. However, much is still uncertain regarding the pathophysiology of HSP [7], and several findings suggest that genetic factors may also have a role to play. The incidence of HSP is highest in people of Asian descent [8], and HSP can occur in siblings [9]. First-degree relatives of HSP patients have elevated levels of serum galactose-deficient IgA, an abnormally glycosylated IgA molecule suggested to participate in the disease pathogenesis [10]. Although no single gene has been found to be causative of HSP, several human leukocyte antigen (HLA) class I and II alleles have been linked to an increased risk of HSP [11]. Most studies evaluating the genetic background of HSP have been candidate gene studies where the analyses focused on one specific gene of interest [11]. Genome-wide association studies (GWAS), on the other hand, provide a more comprehensive and hypothesis-free analysis throughout the genome. However, to date, only one GWAS has been performed in HSP: in a Spanish cohort, HSP was linked to a linkage disequilibrium (LD) block of polymorphisms within HLA class II [12].

This study is aimed at performing a GWAS in childhood-onset HSP patients, with an emphasis on severe HSP nephritis (HSN). Several case reports have also suggested that pediatric inflammatory bowel disease (IBD) patients may develop kidney involvement. The most common co-occurring diagnoses are nephrolithiasis and tubulointerstitial nephritis, but reports have also described the co-existence of IBD and IgA nephropathy (IgAN) [13], which is a disease that shares common pathophysiologic features with HSN [14]. The present study therefore included pediatric IBD patients as an autoimmune disease control group, while Finnish bone marrow and blood donors represented a general reference population.

## Methods

### Study population and controls

The study included 52 subjects with childhood-onset (< 17 years) HSP from five university hospitals in Finland. All patients fulfilled the EULAR/PRINTO/PRES classification criteria for HSP [15]. The analyses also involved 53 childhood-onset (< 17 years) IBD patients with biopsy-proven Crohn’s disease and/or orofacial granulomatosis. Written informed consent was obtained from all participants (and their guardians if necessary). The study was approved by the Ethics Committee of Helsinki University Hospital (application numbers 164/13/03/03/2016, 240/13/03/02/13, and HUS/1176/2016). After quality controls (described below and in detail in Supplementary Note 1), the final study population comprised 46 HSP patients and 49 IBD patients. The reference group representing the Finnish population consisted of (total *n* = 18,757) Finnish bone marrow donors (*n* = 216) and Finnish blood donors (*n* = 18,541).

### Clinical parameters

Medical records provided clinical data until the last follow-up visit for HSP patients and at disease onset for IBD patients. Additionally, to detect possible kidney involvement in IBD patients, laboratory records were reviewed for urine dipstick and microscopy examinations and measurements of urine protein. Bedside Schwartz equation [16] provided estimated glomerular filtration rate values (eGFR). Definition of hematuria was > 20 red blood cells/10^6^/L or at least two consecutive positive dipstick tests (2+ to 3+). Definition of proteinuria was urine protein to creatinine ratio (UP/C) > 20 mg/mmol or at least two consecutive positive dipstick tests (2+ to 3+); a daily urine protein excretion (dU-Prot) > 40 mg/h/m^2^ denoted nephrotic-range proteinuria. If needed, dU-Prot was calculated from UP/C [17]. The presence of hematuria and/or proteinuria denoted kidney involvement. For HSP patients who had undergone kidney biopsy, the International Study of Kidney Disease in Children (ISKDC) grade was obtained from biopsy reports.

Table 1 presents the baseline characteristics of HSP and IBD patients. Forty-four (96%) HSP patients had kidney involvement; 42 of them had undergone a kidney biopsy. The remaining two HSP patients did not develop HSN within a two-month follow-up. Urinary analyses were available from 28 (57%) IBD patients. Abnormal findings in urine dipstick and/or microscopy analyses occurred in four IBD patients (hematuria in three and hematuria + proteinuria in one); in two of them, the urinary findings had normalized in control samples. None of these four IBD patients had undergone a kidney biopsy or been diagnosed with a kidney disease.

**Table 1:** Baseline characteristics of HSP and IBD patients

	HSP (*n* = 46)	IBD (*n* = 49)
Gender	19 boys, 27 girls	33 boys, 16 girls
Age at diagnosis	9.1 (7.3–11.9)	12.9 (9.2–14.3)
Plasma creatinine^a^ (μmol/L)	51 ± 18	-
eGFR^a^ (mL/min/1.73 m^2^)	108 ± 27	-
dU-Prot^a^ (mg/h/m^2^)	102 (37–215)	-
Nephrotic-range proteinuria^a^, n (%)	30 (71)	-
Plasma albumin^a^ (g/L)	29.9 ± 7.7	-
Kidney biopsy findings, *n* (%)		-
ISKDC I	2 (5)	
ISKDC II	6 (14)	
ISKDC III	27 (64)	
ISKDC IV	4 (10)	
ISKDC V	1 (2)	
ISKDC VI	2 (5)	
IBD characteristics, *n* (%)	-	
CD		22 (45)
OFG		8 (16)
CD + OFG		19 (39)

HSP: Henoch-Schönlein purpura; IBD: inflammatory bowel disease; eGFR: estimated glomerular filtration rate; dU-Prot: daily urine protein excretion; ISKDC: International Study of Kidney Disease in Children; CD: Crohn’s disease; OFG: orofacial granulomatosis. ^a^Measured at the kidney biopsy (HSP, *n* = 42)

### Genotyping, quality control, and imputation

Supplementary Note 1 contains a detailed description of the genotyping, quality control, and genotype and HLA imputations. Briefly, blood donor samples were genotyped with FinnGen array, whereas HSP, IBD, and bone marrow donor samples were genotyped with Illumina Global Screening Array (GSA). Data genotyped on the GSA platform underwent a genotype lift-over from hg19 to hg38 [18]. A platform-bias analysis was performed on control samples between the blood and bone marrow donor samples, and single-nucleotide polymorphisms (SNP) associated with the genotyping platform were excluded from the data (Supplementary Figure S1). Plink v. 1.9 [19] provided tools for quality control of HSP, IBD, and reference population data. Exclusion criteria for SNPs were missing call rate > 5%, minor allele frequency < 1%, and Hardy-Weinberg equilibrium probability test score < 1 × 10^-6^. Exclusion criteria for individuals were missing call rate > 10%, those with disturbances in genotyping reports, kinship with other samples (of these, the individual with the greater missing call rate was excluded), or discordant sex information. After variant pruning based on LD, we performed principal component (PC) analyses to detect population stratification within the data and to obtain PCs for association analyses (Supplementary Figure S2). HLA imputation was performed with the R library HIBAG [20] for HLA genes HLA-A, HLA-B, HLA-C, HLA-DRB1, HLA-DQA1, HLA-DQB1, and HLA-DPB1 using an imputation model for the Finnish population [21]. In addition, amino acid sequences were obtained for the imputed HLA alleles using HIBAG [20].

### Statistical analyses

R 3.6.3 (R Foundation for Statistical Computing, Vienna, Austria) provided statistical tools for GWAS and HLA analyses. GWAS was performed using the R package SPAtest [22] for HSP and IBD samples in a case-control manner against the reference population. The first three PCs, sex, and genotyping platform were included as covariates. Manhattan plots were drawn with the R package qqman [23]. An association analysis for HSP and IBD samples against the reference population was performed using dosages of imputed HLA alleles and their amino acid sequences. The R package SPAtest [22] was used for the association analyses with the first three PCs, sex, and genotyping platform as covariates. Genetic correlation analysis between HSP and IBD was performed as described in McGeachie et al. [24]. Results are reported as *p* values and odds ratios (OR) with their 95% confidence intervals (95% CI). Genome-wide level of statistical significance (*p* < 5 × 10^-8^) served as a threshold for statistical significance. We estimated the statistical power of the top genome-wide significant SNPs and HLA alleles with Monte Carlo simulations. A simulation run consisted of sampling randomly 10,000 times from the observed genotype distributions of cases and controls followed by performing the SPA score test as described above for all the samplings. Five normally distributed random variables were included as covariates in the SPA test analysis. Power was determined by calculating the proportion of *p* values below 5 × 10^-8^.

Clinical parameters were analyzed with IBM SPSS version 25 (IBM Corp, Armonk, NY). Normally distributed continuous variables are presented as means and standard deviations (SD) and non-normally distributed continuous variables as medians with interquartile range (IQR). All continuous variables were analyzed with the Mann-Whitney U test. Categorical variables are presented as numbers and percentages, and they were analyzed with Fisher’s exact test. Clinical data analyses applied *p* < 0.05 as a level of significance.

## Results

**Fig. 1:**
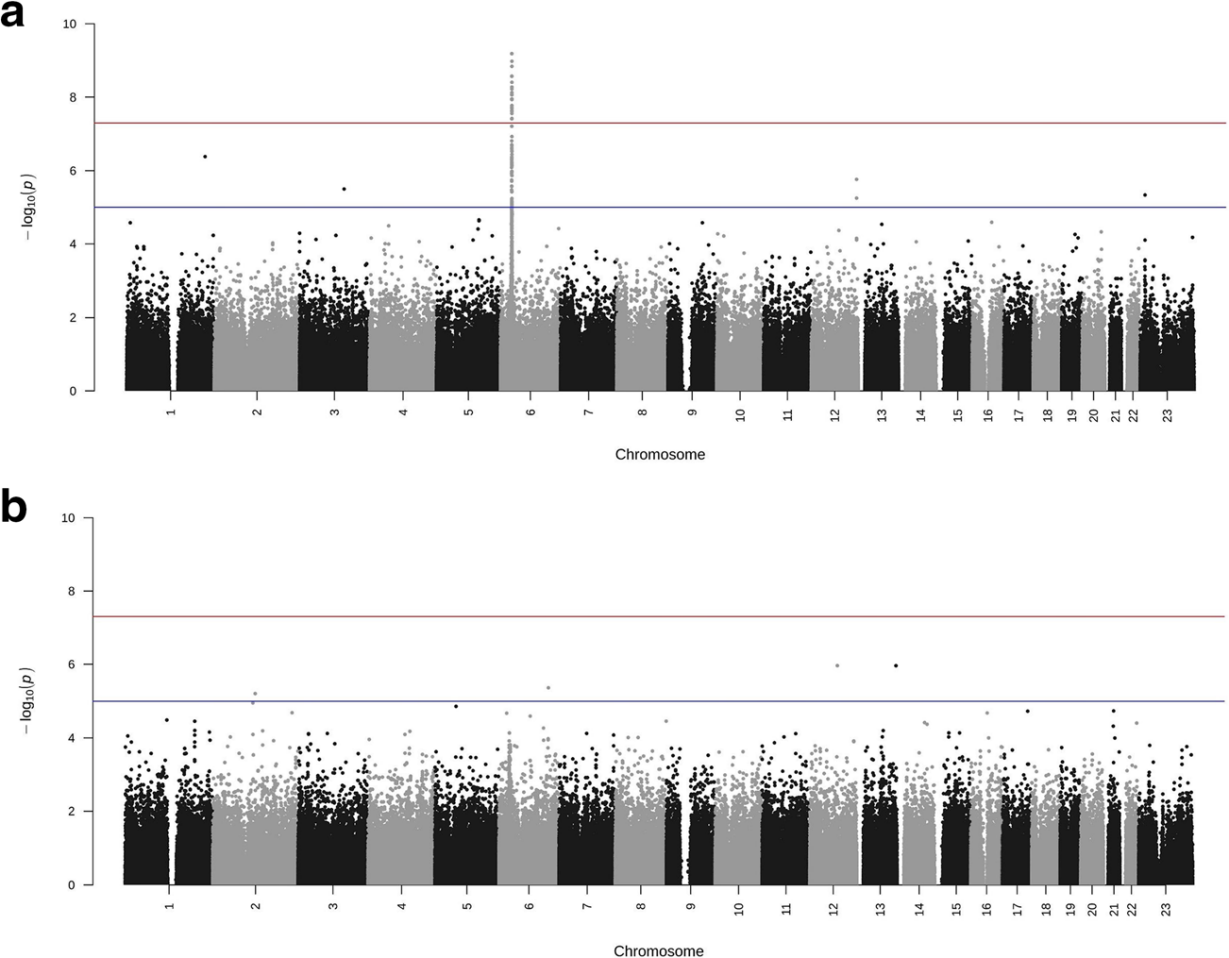
GWAS results in HSP (**a**) and IBD (**b**) illustrated in a Manhattan plot. The red line represents genome-wide significance level (*p* < 5 × 10^-8^) and the blue line significance level of *p* < 1 × 10^-5^

**Fig. 2:**
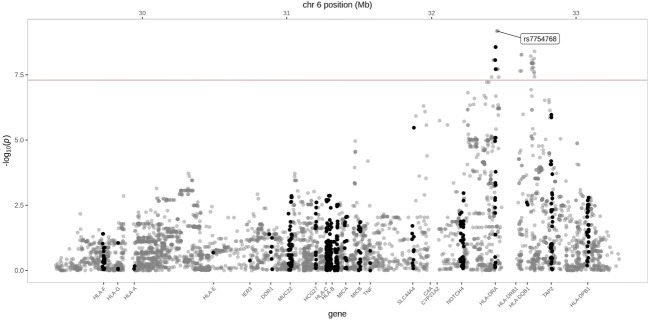
Detailed GWAS results in HSP from HLA region in chromosome 6. The red line represents genome-wide significance level (*p* < 5 × 10^-8^)

Figure 1 illustrates the main results of the GWAS analyses. In HSP, several polymorphisms in the HLA region in chromosome 6 occurred at the genome-wide statistical significance level (Fig. 1a). A list of SNPs with the greatest association with HSP is visible in Supplementary Table S1. In IBD, no polymorphism exhibited significance at the genome-wide level (Fig. 1b). Figure 2 presents more detailed results from the HLA region in HSP.

After HLA imputation, we compared the occurrence of HLA alleles between HSP patients and the reference population. At the genome-wide level of significance, three HLA alleles were more common in HSP than in the reference population: DQA1*01:01, DQB1*05:01, and DRB1*01:01 (Table 2). Of the HLA class I alleles, none appeared significant at the genome-wide level. The strongest associations occurred in alleles C*04:01 (HSP 35% vs. reference population 16%; *p* = 0.0018; OR 2.21, 95% CI 1.70–2.87) and B*35:01 (HSP 30% vs. reference population 13%; *p* = 0.0032; OR 2.15, 95% CI 1.64–2.81). In IBD patients, no HLA class I or II allele differed from the reference population at the genome-wide significance level. The HLA class II alleles with the strongest association with HSP (i.e., DQA1*01:01, DQB1*05:01, and DRB1*01:01) occurred less frequently in IBD patients than in the reference population (Table 2). Supplementary Table S2 provides all HLA alleles associated with HSP and IBD with a significance level of *p* < 0.05. Supplementary Table S3 contains results from the power analyses for the top genome-wide significant SNPs and HLA alleles in HSP (rs7754768, rs9275578, HLA-DQA1*01:01, HLA-DQB1*05:01, and HLA-DRB1*01:01). Power was > 0.9 for all analyzed SNPs and HLA alleles.

**Table 2:** HLA alleles associated with HSP at the genome-wide level of significance when compared against the reference population. In addition, the table provides association of the same alleles between IBD patients and the reference population

	HSP (*n* = 46)			IBD (*n* = 49)			Reference population (*n* = 18,757)
HLA allele	*p* value	OR [95% CI]	Allele frequency	*p* value	OR [95% CI]	Allele frequency	Allele frequency
DQB1*05:01	4.99 × 10^-09^	4.37 [3.35–5.71]	0.58	0.0016	0.36 [0.26–0.51]	0.11	0.20
DQA1*01:01	1.04 × 10^-08^	4.18 [3.21–5.45]	0.55	0.0014	0.35 [0.24–0.49]	0.10	0.19
DRB1*01:01	2.37 × 10^-08^	4.10 [3.14–5.35]	0.54	0.0013	0.34 [0.24–0.49]	0.10	0.19

HSP: Henoch-Schönlein purpura; IBD: inflammatory bowel disease; HLA: human leukocyte antigen

Haplotype DQA1*01:01/DQB1*05:01/DRB1*01:01 occurred in 20 (43.5%) HSP patients, in 4 (8.2%) IBD patients, and in 15.0% of the reference population (HSP vs. reference population; *p* = 1.8 × 10^-8^; OR 4.15, 95% CI 3.18–5.41). In addition, the haplotype B*35:01/DQA1*01:01/DQB1*05:01/DRB1*01:01 occurred in 14 (30.4%) HSP patients, in 1 (2.0%) IBD patient, and in 5.4% of the reference population (HSP vs. reference population; *p* = 0.0015; OR 2.28, 95% CI 1.74–2.99). To evaluate whether haplotype DQA1*01:01/DQB1*05:01/DRB1*01:01 or B*35:01/DQA1*01:01/DQB1*05:01/DRB1*01:01 associated with a more severe form of HSN, we compared initial clinical findings and outcome in HSP patients with and without the haplotypes. Detailed results of the comparisons are visible in Supplementary Tables S4 and S5. HSP patients positive for haplotype DQA1*01:01/DQB1*05:01/DRB1*01:01 or B*35:01/DQA1*1:01/DQB1*05:01/DRB1*01:01 showed no significant difference in age, gender, eGFR, proteinuria levels, kidney biopsy findings, or outcomes compared to those negative for the haplotypes.

To evaluate whether HSN and IBD share any common genetic variability, we performed a genetic correlation analysis between HSP and IBD patients. We found a significant genetic component between the two diseases. Supplementary Table S6 provides SNPs that occurred in both diseases within the HLA region. The common SNPs between the diseases are located on class I gene *HLA-B* and on class II genes *HLA-DRB1* and *HLA-DQB1*.

## Discussion

The present study represents a GWAS in patients with childhood-onset HSP. Our results, consistent with previous findings [12], suggest that particularly specific HLA region II alleles may have a significant association with HSP susceptibility. Haplotype DQA1*01:01/DQB1*05:01/DRB1*01:01 occurred in 43.5% of HSP patients, while it was present in only 15.0% of the reference population. Also, haplotype B*35:01/DQA1*01:01/DQB1*05:01/DRB1*01:01 differed between HSP patients (30.4%) and the reference population (5.4%). No significant associations occurred outside the HLA region.

To our knowledge, in HSP, only one previous GWAS exists, conducted by López-Mejías et al., including HSP patients of Spanish descent [12]. There are some differences in the study population between our cohort and the Spanish GWAS cohort. For example, the Spanish cohort also included adult patients (19% of the cohort) and had fewer patients with kidney involvement (38%) and nephrotic syndrome (4%). Polymorphisms with the strongest association with HSP in the study by López-Mejías et al. occurred at an intergenic region between *HLA-DQA1* and *HLA-DQB1*, which, in turn, is high in LD with the *HLA-DRB1* gene. A potential signal also occurred in the *HLA-B* gene [12]. These findings are consistent with those of our study. Given the differences in the study population compared to our cohort, these observed HLA associations may therefore reflect susceptibility to HSP and not to HSN or other HSP subgroup. This notion is further supported by the finding that in our study, patients with haplotype DQA1*01:01/DQB1*05:01/DRB1*01:01 or B*35:01/DQA1*01:01/DQB1*05:01/DRB1*01:01 had similar initial kidney findings and outcome as those negative for the haplotypes.

Consistent with our findings, previous studies of Italian and Spanish HSP cohorts have reported an association with HLA-DRB1*01 allele and susceptibility to HSP [25–27]. However, contrary findings were made in studies performed in Turkish [28] and Indian cohorts [29]. Studies have also reported associations with HLA-DRB1*11 [26, 28, 29] and HLA-DRB1*14 [28]. Interestingly, all studies reported no HLA-DRB1 phenotype differences between patients with and without kidney involvement [25–29], although in the study by Soylemezoglu et al. HLA-DRB1*13 occurred more frequently in patients with nephrotic proteinuria [28]. It thus seems that the association with HLA-DRB1 is independent of the kidney component of HSP, but it remains unclear whether susceptibility to HSP is dependent on the analyzed population. Few studies have evaluated the role of other HLA class II genes in HSP. In the study by Amoroso et al., HLA-DQA1*0101, HLA-DQB1*0501, and HLA-DQB1*0301 occurred more frequently in HSP patients [26]. Jin et al. also reported a possible association with HLA-DQA1*0301 in Korean HSP patients [30].

For the HLA class I region, earlier studies have shown mixed results. In our study, of the class I alleles, HLA-C*04:01 and HLA-B*35:01 exhibited the greatest association with HSP, but nonetheless, they did not reach a genome-wide level of significance. HLA-B*35 has been previously implicated to cause HSP susceptibility in some cohort studies [31, 32], whereas others have failed to validate this association [33–35]. An Italian study also suggested that HLA-B*35 was associated with HSN [33], but subsequent and larger cohort studies have shown contradictory results [32, 34]. A study on Spanish HSP patients also suggested that HLA-B*41:02 is associated with disease susceptibility [34]. In addition to HLA-B alleles, a Turkish study suggested that HLA-A*2 and HLA-A*11 may be associated with predisposition to HSP [32].

GWAS and HLA imputation studies in IgAN have emphasized the importance of the HLA region and revealed several risk and protective SNPs and HLA alleles [36]. They have occurred mainly in the HLA-DQ region, and some of the risk alleles in our HSP samples have also been linked with an increased risk in IgAN. These include DQA1*01:01 in a cohort of European and East Asian patients [37] and DQB1*05:01 in British patients [38]. Other immune-mediated kidney diseases, such as anti-neutrophil cytoplasmic antibody (ANCA)-associated vasculitis, have also shown HLA associations, but the HLA alleles associated with increased risk vary between diseases [36].

To evaluate whether the obtained results reflect a general susceptibility to autoimmune diseases, our study also included pediatric IBD patients. Extraintestinal manifestations occur in 20–35% of children with Crohn’s disease, but the prevalence of kidney involvement in pediatric IBD is unclear [13]. However, a recent study suggested that the incidence of IgAN is elevated in adult IBD patients [39]. The connection between IgAN and IBD was further supported by a GWAS in patients with IgAN, which revealed several potential susceptibility loci to IgAN that are also associated with the risk of IBD, are involved in intestinal mucosal barrier maintenance, or regulate mucosal immune response [37]. In our study, HLA status in IBD patients was for the most part dissimilar to that of HSP patients. Nonetheless, there were some possible common SNPs between the diseases in *HLA-B*, *HLA-DRB1*, and *HLA-DQB1* genes. Their clinical interpretation is, however, unclear as the HLA alleles that are associated with increased susceptibility to HSP (DQA1*01:01, DQB1*05:01, and DRB1*01:01) were protective alleles in IBD patients. As kidney symptoms in our IBD patients were rare, it remains unknown whether IBD patients with evident kidney involvement would share more genetic variability with HSN patients.

One obvious limitation of our study is the limited number of HSP patients. Most HSP patients in the present series had nephrotic-range proteinuria or advanced kidney histology findings; factors that can be characterized as severe kidney involvement. These findings are, however, rare, affecting only a small minority of all HSP patients [3, 40]. Due to the fact that the majority of our patients had HSN, it is possible that the present findings may not be generalizable to Finnish HSP patients without HSN. The small sample size warrants cautious interpretation of the GWAS results. However, our study includes a well-characterized study population and a large reference population representing the Finnish population. The main finding in the GWAS analysis occurred in the HLA region, which is consistent with earlier studies [12, 25–27]. In addition, despite the small sample size, the power analyses support the reliability of the present results.

In conclusion, our results suggest that HLA class II alleles are associated with susceptibility to HSP, mainly due to DQA1*01:01/DQB1*05:01/DRB1*01:01 haplotype. However, this haplotype was not associated with the severity of kidney involvement. Possible signals occurred within HLA class I as well. These HLA associations did not occur in IBD patients, suggesting that they are specific to HSP and not related to susceptibility to autoimmune diseases in general.

## Supplementary Information

ESM 1 (PDF 573 kb).

ESM 2 (PDF 857 kb).

ESM 3 (PDF 561 kb).

ESM 4 (PPTX 204 kb).

## Data Availability

The datasets generated during and/or analyzed during the current study are available from the corresponding author on reasonable request.
