# Supplementary material for: HLA-DQ and HLA-DRB1 alleles associated with Henoch-Schönlein purpura nephritis in Finnish pediatric population: a genome-wide association study
Source: Pediatr Nephrol. 2021 Feb 16;36(8):2311–8. doi: 10.1007/s00467-021-04955-7 (PMC8260528; doi:10.1007/s00467-021-04955-7)
Supplement: Supplementary file 1 — (PDF 573 kb). [file 467_2021_4955_MOESM1_ESM.pdf]

Supplementary Note 1

Article title: HLA-DQ and HLA-DRB1 alleles associated with Henoch-Schönlein purpura nephritis in Finnish pediatric population: A genome-wide association study

Journal: Pediatric Nephrology

Authors: Mikael Koskela*, Julia Nihtilä*, Elisa Ylinen, Kaija-Leena Kolho, Matti Nuutinen, Jarmo Ritari, Timo Jahnukainen. *Contributed equally to this work

Corresponding author: Mikael Koskela; Children's Hospital, Pediatric Research Center, University of Helsinki, Helsinki University Hospital, Helsinki, Finland. e-mail address: mikael.koskela@helsinki.fi

Methods

Quality control

Quality control was performed for HSP, IBD, HSCT and BD data using Plink v1.9 (Chang et al., 2015) and SNPs with missing call rate > 5%, minor allele frequency (MAF) < 1%, or Hardy-Weinberg equilibrium probability test score < 1E-6 were excluded along with individuals with missing call rates > 10%, and individuals mentioned having disturbances in genotyping reports. Two HSP samples were excluded based on their HSP status. Sex information was checked for HSP, IBD, and HSCT data, and imputed for BD data using Plink v1.9 (Chang et al., 2015). Individuals with discordant sex information or failed sex imputation were excluded from the data.

Genotype lift-over from hg19 to hg38

HSP, IBD, and HSCT data genotyped on GSA platform was subjected to a genotype lift-over from hg19 to hg38 according to Genotyping chip data lift-over to reference genome build GRCh38/hg38 V.2 protocol (Pärn Kalle et al., 2019). Prior to this, duplicate variants had been removed from the data using Plink v1.9 and R (Chang et al., 2015; R Core Team, 2020). Allele frequency files prepared for EUR samples of the 1000 Genomes Project GRCh38/hg38 data included in the protocol were used as reference allele frequency data, and the strand file used in the protocol containing the names and new locations of the SNPs was modified to match SNP naming in HSP, IBD, and HSCT data with R (R Core Team, 2020).

Overall, 448,657 SNPs were lifted over to hg38 (99.6%) including 435,289 SNPs with concordant allele frequencies to the reference allele frequencies and 13,368 SNPs with a high allele frequency difference, likely due to the reference population being European and not Finnish.

LD pruning

Variant pruning based on linkage disequilibrium was conducted on the data using Plink v1.9 (Chang et al., 2015) in order to obtain suitable data for principal component analysis and identity-by-descent analysis. Parameters for pruning were window size of 50 variants, variant count of 5 to shift the window, and r^2 threshold of 0.5.

PCA

Principal component analysis was performed on LD pruned data using Plink v1.9 (Chang et al., 2015) intending to detect population stratification within the data and to obtain principal components to be used in association analysis. Plotting the PCA results with R (R Core Team) revealed zero outliers, so no individuals were excluded from the data. The first three principal components were selected as covariates from a PCA conducted to the final dataset after all quality control.

IBD analysis

Identity-by-descent analysis was conducted on LD pruned data as a means to take account of kinship in the upcoming genome-wide association study. The analysis was performed using Plink v1.9 (Chang et al., 2015) with the parameter min 0.2. In total, 1035 pairs of individuals were first-degree relatives (IBD > 0.4) and the individual with greater missingness was excluded from each pair.

Platform bias analysis

A platform-bias analysis was performed on controls genotyped on different platforms (HSCT samples on GSA platform and BD samples on FinnGen array) as a genome-wide association study of HSCT samples against BD samples. R package SPAtest (Dey et al., 2017) was used for this, and SNPs associating with GSA platform (p < 1E-4, in total) were excluded from the data. The first three principal components and sex were used as covariates.

HLA imputation and obtaining protein sequences for imputed alleles

Imputing HLA genotypes (for HLA genes HLA-A, HLA-B, HLA-C, HLA-DRB1, HLA-DQA1, HLA-DQB1, and HLA-DPB1) for the data was executed using previously-made imputation models for Finnish samples (Ritari et al., 2020). The MHC region (chromosome 6 28-34MB) had been extracted into a separate data set for this beforehand with Plink v1.9 (Chang et al., 2015), and R package HIBAG was used for the imputation (Zheng et al., 2014).

Amino acid sequences were obtained for the imputed HLA alleles using the R package HIBAG (Zheng et al., 2014), and a dosage table was created for association analysis with R (R Core Team, 2020) of both HLA alleles and their amino acid sequences excluding amino acids showing little to no variation (amino acids identical to reference sequence, for instance).

GWAS

A genome-wide association study was performed both for HSP and IBD samples in a case - control manner against HSCT and blood donor controls. R package SPAtest (Dey et al., 2017) was used for this utilizing the first three principal components, sex and information on genotyping platform as covariates. Manhattan plots were drawn of the results with the R package qqman (Turner, 2018).

Association analysis for imputed HLA alleles and HLA protein sequences

An association study was performed for the imputed HLA genotypes and their amino acid sequences in a case - control manner both for HSP and IBD samples against common HSCT and blood donor controls using dosage tables constructed while imputing HLA genotypes and their amino acid sequences. The R package SPAtest (Dey et al., 2017) was used for the association analysis, with the first three principal components, sex and genotyping platform as covariates.

References:

Chang, C.C., Chow, C.C., Tellier, L.C., Vattikuti, S., Purcell, S.M., and Lee, J.J. (2015). Second-generation PLINK: rising to the challenge of larger and richer datasets. *Gigascience 4,7*

Dey, R., Schmidt, E.M., Abecasis, G.R., and Lee, S. (2017). A Fast and Accurate Algorithm to Test for Binary Phenotypes and Its Application to PheWAS. *Am. J. Hum. Genet. 101,* 37-49.

Pärn Kalle, Fontarnau Javier Nunez, Isokallio Marita, A., Sipilä Timo, Kilpelainen Elina, Palotie Aarno, Ripatti Samuli, and Palta Priit. (2019). Genotyping chip data lift-over to reference genome build GRCh38/hg38 V.2.*V.2,* https://www.protocols.io/view/genotyping-chip-data-lift-over-to-reference-genome-xbhfij6?step=6

R Core Team. (2020). R: A language and environment for statistical computing. *R package version 3.6.3.*

Ritari J, Hyvärinen K, Clancy J, Partanen J, Koskela S. Increasing accuracy of HLA imputation by a population-specific reference panel in a FinnGen biobank cohort. *NAR: Genomics and Bioinformatics.* 2020 2(2).

Turner, S.D. (2018). qqman: an R package for visualizing GWAS results using Q-Q and manhattan plots. *Journal of Open Source Software 3,* 731-732.

Zheng, X., Shen, J., Cox, C., Wakefield, J.C., Ehm, M.G., Nelson, M.R., and Weir, B.S. (2014). HIBAG—HLA genotype imputation with attribute bagging. *The Pharmacogenomics Journal* 14, 192-200.
