# Supplementary material for: HLA-DQ and HLA-DRB1 alleles associated with Henoch-Schönlein purpura nephritis in Finnish pediatric population: a genome-wide association study
Source: Pediatr Nephrol. 2021 Feb 16;36(8):2311–8. doi: 10.1007/s00467-021-04955-7 (PMC8260528; doi:10.1007/s00467-021-04955-7)

Figure S1

Article title: HLA-DQ and HLA-DRB1 alleles associated with Henoch-Schönlein purpura nephritis in Finnish pediatric population: A genome-wide association study
Journal: Pediatric Nephrology
Authors: Mikael Koskela*, Julia Nihtilä*, Elisa Ylinen, Kaija-Leena Kolho, Matti Nuutinen, Jarmo Ritari, Timo Jahnukainen.
*Contributed equally to this work
Corresponding author: Mikael Koskela; Children's Hospital, Pediatric Research Center, University of Helsinki, Helsinki University Hospital, Helsinki, Finland. e-mail address: mikael.koskela@helsinki.fi

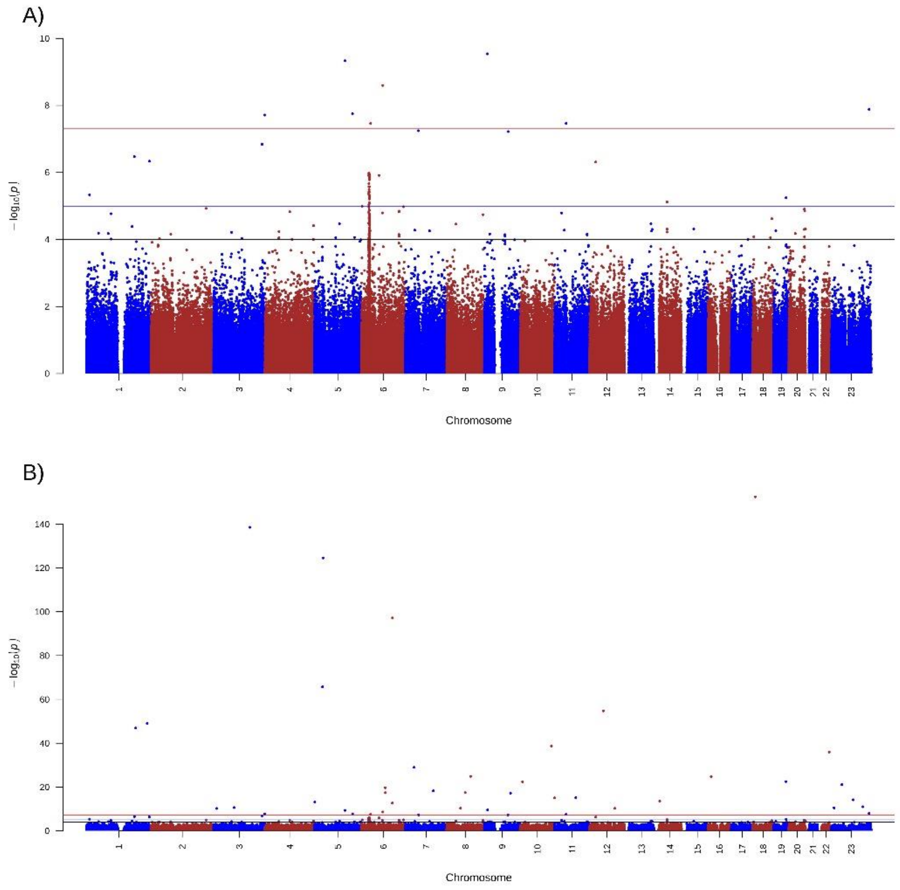

Figure S2

Article title: HLA-DQ and HLA-DRB1 alleles associated with Henoch-Schönlein purpura nephritis in Finnish pediatric population: A genome-wide association study
Journal: Pediatric Nephrology
Authors: Mikael Koskela*, Julia Nihtilä*, Elisa Ylinen, Kaija-Leena Kolho, Matti Nuutinen, Jarmo Ritari, Timo Jahnukainen.
*Contributed equally to this work
Corresponding author: Mikael Koskela; Children's Hospital, Pediatric Research Center, University of Helsinki, Helsinki University Hospital, Helsinki, Finland. e-mail address: mikael.koskela@helsinki.fi

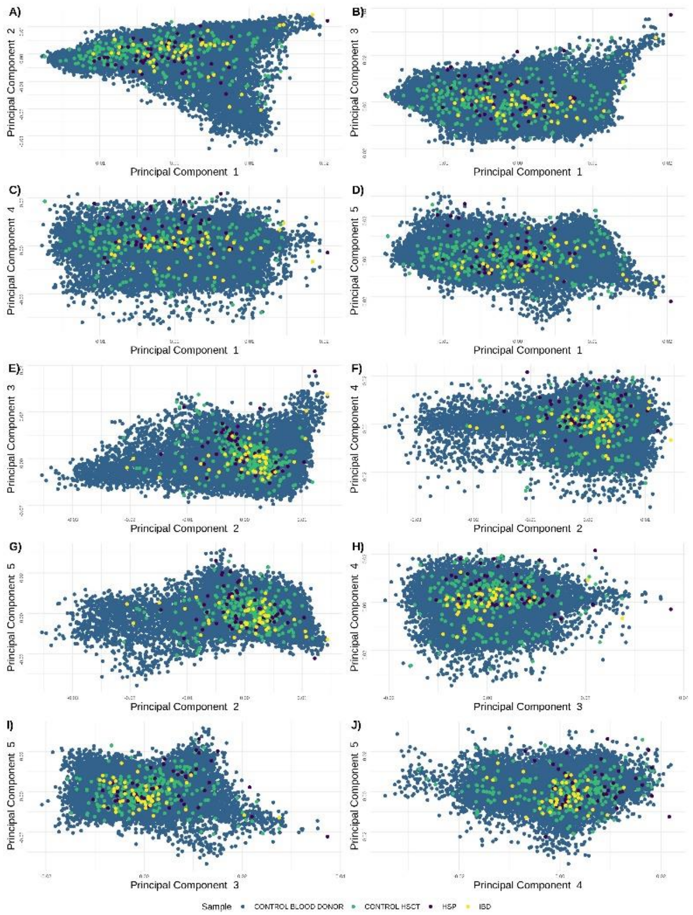

Supplement: Supplementary file 2 — (PDF 857 kb). [file 467_2021_4955_MOESM2_ESM.pdf]
