# Supplementary material for: HLA-DQ and HLA-DRB1 alleles associated with Henoch-Schönlein purpura nephritis in Finnish pediatric population: a genome-wide association study
Source: Pediatr Nephrol. 2021 Feb 16;36(8):2311–8. doi: 10.1007/s00467-021-04955-7 (PMC8260528; doi:10.1007/s00467-021-04955-7)
Supplement: Supplementary file 3 — (PDF 561 kb). [file 467_2021_4955_MOESM3_ESM.pdf]

**Article title:** HLA-DQ and HLA-DRB1 alleles associated with Henoch-Schönlein purpura nephritis in Finnish pediatric population: A genome-wide association study

**Journal:** Pediatric Nephrology

**Authors:** Mikael Koskela*, Julia Nihtilä*, Elisa Ylinen, Kaija-Leena Kolho, Matti Nuutinen, Jarmo Ritari, Timo Jahnukainen. *Contributed equally to this work

**Corresponding author:** Mikael Koskela; Children's Hospital, Pediatric Research Center, University of Helsinki, Helsinki University Hospital, Helsinki, Finland. e-mail address: mikael.koskela@helsinki.fi

**Table S1.** Polymorphisms associated to HSP susceptibility in GWAS at the genome-wide significance level $p < 5 \times 10^{-08}$

| SNP | Position | Minor allele | p-value | OR [95% CI] | Frequency HSP | Frequency reference population |
|---|---|---|---|---|---|---|
| rs7754768 | 32.452.402 | C | 6.57e-10 | 0.14 [0.10-0.20] | 0.14 | 0.49 |
| rs9268832 | 32.460.012 | T | 6.57e-10 | 0.14 [0.10-0.20] | 0.14 | 0.49 |
| rs9275578 | 32.711.607 | T | 1.06e-09 | 4.62 [3.51-6.10] | 0.71 | 0.31 |
| rs9275582 | 32.712.293 | T | 1.46e-09 | 4.54 [3.45-5.98] | 0.71 | 0.31 |
| rs2213585 | 32.445.373 | G | 2.71e-09 | 0.15 [0.11-0.22] | 0.14 | 0.48 |
| rs2213586 | 32.445.317 | A | 2.71e-09 | 0.15 [0.11-0.22] | 0.14 | 0.49 |
| rs2227139 | 32.445.682 | G | 2.71e-09 | 0.15 [0.11-0.22] | 0.14 | 0.49 |
| rs3763327 | 32.446.053 | G | 2.71e-09 | 0.15 [0.11-0.22] | 0.14 | 0.49 |
| rs7192 | 32.443.869 | T | 2.71e-09 | 0.15 [0.11-0.22] | 0.14 | 0.49 |
| rs7195 | 32.444.762 | A | 2.71e-09 | 0.15 [0.11-0.22] | 0.14 | 0.49 |
| rs3998158 | 32.714.215 | C | 3.97e-09 | 4.39 [3.33-5.79] | 0.70 | 0.30 |
| rs112396255 | 32.623.692 | G | 5.35e-09 | 4.50 [3.42-5.91] | 0.58 | 0.22 |
| rs17533090 | 32.622.945 | T | 5.35e-09 | 4.50 [3.42-5.91] | 0.58 | 0.22 |
| rs13194877 | 32.687.771 | A | 6.06e-09 | 4.48 [3.41-5.89] | 0.57 | 0.20 |
| rs9275572 | 32.711.222 | A | 7.58e-09 | 0.17 [0.12-0.24] | 0.13 | 0.48 |
| rs17202717 | 32.430.086 | T | 8.59e-09 | 4.33 [3.31-5.67] | 0.57 | 0.20 |
| rs17496307 | 32.433.259 | T | 8.59e-09 | 4.33 [3.31-5.67] | 0.57 | 0.20 |
| rs17496549 | 32.441.931 | T | 8.59e-09 | 4.33 [3.31-5.67] | 0.57 | 0.20 |
| rs17212483 | 32.697.473 | T | 8.78e-09 | 4.27 [3.27-5.58] | 0.57 | 0.20 |
| rs115255544 | 32.696.851 | G | 1.14e-08 | 4.23 [3.24-5.52] | 0.57 | 0.20 |
| rs17212462 | 32.697.348 | T | 1.14e-08 | 4.23 [3.24-5.52] | 0.57 | 0.20 |
| rs17212553 | 32.697.910 | T | 1.14e-08 | 4.23 [3.24-5.52] | 0.57 | 0.20 |
| rs17427445 | 32.695.987 | A | 1.14e-08 | 4.23 [3.24-5.52] | 0.57 | 0.20 |
| rs17427564 | 32.699.290 | C | 1.14e-08 | 4.23 [3.24-5.52] | 0.57 | 0.20 |
| rs34492195 | 32.703.056 | A | 1.14e-08 | 4.23 [3.24-5.52] | 0.57 | 0.20 |
| rs34677551 | 32.696.159 | C | 1.14e-08 | 4.23 [3.24-5.52] | 0.57 | 0.20 |
| rs35472814 | 32.692.120 | C | 1.14e-08 | 4.23 [3.24-5.52] | 0.57 | 0.20 |
| rs35900855 | 32.696.261 | G | 1.14e-08 | 4.23 [3.24-5.52] | 0.57 | 0.20 |
| rs9275516 | 32.706.866 | A | 1.14e-08 | 0.18 [0.13-0.25] | 0.13 | 0.48 |

| | | | | | | |
|---|---|---|---|---|---|---|
| rs10484561 | 32.697.643 | G | 1.67e-08 | 4.22 [3.22-5.52] | 0.57 | 0.20 |
| rs35332745 | 32.706.448 | C | 1.67e-08 | 4.11 [3.16-5.35] | 0.57 | 0.20 |
| rs12529093 | 32.448.081 | G | 1.91e-08 | 4.13 [3.16-5.38] | 0.58 | 0.21 |
| rs13209059 | 32.448.301 | G | 1.91e-08 | 4.13 [3.16-5.38] | 0.58 | 0.21 |
| rs13209234 | 32.448.198 | A | 1.91e-08 | 4.13 [3.16-5.38] | 0.58 | 0.21 |
| rs13220657 | 32.456.254 | T | 1.91e-08 | 4.13 [3.16-5.38] | 0.58 | 0.21 |
| rs28895028 | 32.447.434 | A | 1.91e-08 | 4.13 [3.16-5.38] | 0.58 | 0.21 |
| rs28895205 | 32.457.527 | G | 1.91e-08 | 4.13 [3.16-5.38] | 0.58 | 0.21 |
| rs28732229 | 32.459.604 | T | 1.91e-08 | 4.13 [3.16-5.38] | 0.58 | 0.21 |
| rs28895136 | 32.453.727 | C | 1.91e-08 | 4.13 [3.16-5.38] | 0.58 | 0.21 |
| rs3177928 | 32.444.658 | A | 1.91e-08 | 4.13 [3.16-5.38] | 0.58 | 0.21 |
| rs114169440 | 32.695.144 | A | 1.99e-08 | 4.08 [3.13-5.31] | 0.57 | 0.20 |
| rs17599077 | 32.623.281 | C | 2.25e-08 | 4.08 [3.13-5.32] | 0.54 | 0.19 |
| rs4530903 | 32.614.112 | T | 2.25e-08 | 4.08 [3.13-5.32] | 0.54 | 0.19 |
| rs6932517 | 32.710.405 | C | 2.41e-08 | 0.19 [0.14-0.27] | 0.13 | 0.48 |
| rs9275580 | 32.711.685 | G | 2.73e-08 | 3.96 [3.03-5.19] | 0.72 | 0.33 |
| rs2858331 | 32.713.500 | G | 3.74e-08 | 3.82 [2.93-4.97] | 0.65 | 0.33 |
| rs113585425 | 32.465.348 | C | 3.83e-08 | 4.04 [3.10-5.28] | 0.53 | 0.19 |
| rs72847996 | 32.417.115 | A | 3.83e-08 | 4.04 [3.10-5.28] | 0.53 | 0.19 |

**Table S2.** HLA alleles associated with HSP and IBD with a significance level of $p < 0.05$

| | HSP (n = 46) | | | IBD (n = 49) | | | Reference population (n = 18 757) |
|---|---|---|---|---|---|---|---|
| HLA allele | p-value | OR [95% CI] | Allele frequency | p-value | OR [95% CI] | Allele frequency | Allele frequency |
| DQB1*05:01 | $4.99 \times 10^{-09}$ | 4.37 [3.35 – 5.71] | 0.58 | 0.0016 | 0.36 [0.26 – 0.51] | 0.11 | 0.2 |
| DQA1*0101 | $1.04 \times 10^{-08}$ | 4.18 [3.21 – 5.45] | 0.55 | 0.0014 | 0.35 [0.24 – 0.49] | 0.10 | 0.19 |
| DRB1*01:01 | $2.37 \times 10^{-08}$ | 4.10 [3.14 – 5.35] | 0.54 | 0.0013 | 0.34 [0.24 – 0.49] | 0.10 | 0.19 |
| DQA1*05:01 | 0.0013 | 0.04 [0.01 – 0.18] | 0 | - | - | - | 0.10 |
| C*04:01 | 0.0018 | 2.21 [1.70 – 2.87] | 0.35 | - | - | - | 0.16 |
| DQB1*02:01 | 0.0020 | 0.05 [0.01 – 0.20] | 0 | - | - | - | 0.10 |
| DRB1*03:01 | 0.0020 | 0.05 [0.01 – 0.20] | 0 | - | - | - | 0.10 |
| B*35:01 | 0.0032 | 2.15 [1.64 – 2.81] | 0.30 | 0.039 | 0.50 [0.36 – 0.72] | 0.10 | 0.13 |
| DPB1*04:02 | 0.0053 | 2.13 [1.61 – 2.81] | 0.37 | - | - | - | 0.20 |
| DQA1*04:01 | 0.0056 | 0.21 [0.11 – 0.40] | 0.02 | - | - | - | 0.09 |
| DQB1*04:02 | 0.0073 | 0.22 [0.12 – 0.43] | 0.02 | - | - | - | 0.09 |
| DQA1*01:02 | 0.0080 | 0.33 [0.21 – 0.52] | 0.07 | - | - | - | 0.19 |
| DRB1*08:01 | 0.0093 | 0.27 [0.15 – 0.48] | 0.03 | - | - | - | 0.09 |
| C*12:03 | 0.015 | 6.73 [2.85 – 15.88] | 0.04 | 0.00036 | 12.27 [5.75 – 26.21] | 0.06 | 0.04 |
| DPB1*01:01 | 0.018 | 0.09 [0.02 – 0.36] | 0 | - | - | - | 0.06 |
| DRB1*13:01 | 0.019 | 0.29 [0.16 – 0.53] | 0.03 | - | - | - | 0.10 |
| A*25:01 | 0.022 | 7.84 [2.66 – 23.1] | 0.03 | 0.017 | 12.67 [4.15 – 38.73] | 0.02 | 0.01 |
| B*08:01 | 0.024 | 0.27 [0.14 – 0.52] | 0.02 | - | - | - | 0.09 |
| DRB1*04:04 | 0.025 | 3.12 [1.84 – 5.27] | 0.09 | - | - | - | 0.04 |
| A*02:01 | 0.033 | 0.56 [0.43 – 0.74] | 0.25 | - | - | - | 0.33 |
| C*07:02 | 0.034 | 0.44 [0.29 – 0.67] | 0.08 | - | - | - | 0.15 |
| B*44:02 | 0.038 | 2.51 [1.57 – 4.00] | 0.10 | - | - | - | 0.06 |
| DQB1*06:02 | 0.042 | 0.42 [0.27 – 0.66] | 0.07 | - | - | - | 0.15 |

| | | | | | | | |
|---|---|---|---|---|---|---|---|
| DQA1*01:03 | 0.044 | 0.36 [0.2 – 0.63] | 0.03 | - | - | - | 0.09 |
| DQB1*06:03 | 0.044 | 0.36 [0.2 – 0.63] | 0.03 | - | - | - | 0.09 |
| A*32:01 | - | - | - | 0.00089 | 5.5 [3.18 – 9.50] | 0.08 | 0.03 |
| B*39:01 | - | - | - | 0.0081 | 3.92 [2.27 – 6.76] | 0.07 | 0.04 |
| C*02:02 | - | - | - | 0.0098 | 3.06 [1.95 – 4.78] | 0.10 | 0.06 |
| DPB1*17:01 | - | - | - | 0.020 | 94.59 [23.61 – 378.9] | 0.02 | 0 |

**Table S3.** Power analysis results for top genome-wide significant SNPs and HLA alleles in HSP

| SNP or HLA allele | Power |
|---|---|
| rs7754768 | 0.92 |
| rs9275578 | 0.99 |
| DQA1*01:01 | 0.97 |
| DQB1*05:01 | 0.98 |
| DRB1*01:01 | 0.96 |

**Table S4.** HSP data according to DQB105:01/DQA101:01/DRB1*01:01 haplotype

| | **Other haplotypes** | **DQB1*05:01/DQA1*01:01 /DRB1*01:01** | **p-value** |
|---|---|---|---|
| Number of patients | 26 | 20 | |
| Gender (male:female) | 10:16 | 9:11 | 0.77 |
| Renal involvement, n (%) | 25 (96) | 19 (95) | |
| At the renal biopsy[a] | | | |
| Age (years) | 9.8 ± 3.6 | 9.8 ± 2.7 | 0.98 |
| Plasma creatinine (μmol/L) | 51 ± 21 | 51 ± 15 | 0.81 |
| eGFR (mL/min/1.73m$^2$) | 110 ± 28 | 107 ± 27 | 0.48 |
| dU-Prot (g/day) | 3.2 (1.6 – 6.3) | 1.9 (0.9 – 4.9) | 0.36 |
| dU-Prot (mg/h/m$^2$) | 105 (66 – 242) | 83 (31 – 176) | 0.38 |
| Nephrotic-range proteinuria, n (%) | 18 (78) | 12 (63) | 0.32 |
| Plasma albumin (g/L) | 30.4 ± 8.9 | 29.2 ± 6.2 | 0.39 |
| ISKDC ≥ III, n (%) | 17 (74) | 17 (89) | 0.26 |
| At last assessment[b] | | | |
| Proteinuria[c], n (%) | 5 (20) | 7 (37) | 0.31 |
| Follow-up (years) | 8.3 (5.4 – 13.6) | 9.9 (6.9 – 15.6) | 0.36 |

[a]n=42

[b]n=44 (includes patients with HSN; two patients without renal involvement were excluded)

[c]Defined as urine protein to creatinine ratio > 20 mg/mmol or positive dipstick test (1+ to 3+)

**Table S5.** HSP data according to B35:01/DQB105:01/DQA101:01/DRB1*01:01 haplotype

| | **Other haplotypes** | **B*35:01/DQB1*05:01/ DQA1*01:01/DRB1*01:01** | **p-value** |
|---|---|---|---|
| Number of patients | 32 | 14 | |
| Gender (male:female) | 14:18 | 5:9 | 0.75 |
| Renal involvement, n (%) | 31 (97) | 13 (93) | |
| At the renal biopsy[a] | | | |
| Age (years) | 9.8 ± 3.4 | 9.9 ± 2.8 | 0.91 |
| Plasma creatinine (µmol/L) | 50 (40 – 54) | 44 (40 – 65) | 0.91 |
| eGFR (mL/min/1.73m$^2$) | 109 ± 26 | 107 ± 31 | 0.81 |
| dU-Prot (g/day) | 3.2 (1.0 – 6.6) | 1.9 (1.3 – 4.8) | 0.60 |
| dU-Prot (mg/h/m$^2$) | 104 (40 – 250) | 83 (37 – 162) | 0.54 |
| Nephrotic-range proteinuria, n (%) | 21 (72) | 9 (69) | >0.99 |
| Plasma albumin (g/L) | 30.2 ± 9.0 | 29.2 ± 4.4 | 0.43 |
| ISKDC ≥ III, n (%) | 22 (76) | 12 (92) | 0.40 |
| At last assessment[b] | | | |
| Proteinuria[c], n (%) | 6 (19) | 6 (46) | 0.14 |
| Follow-up (years) | 7.7 (4.5 – 13.6) | 12.0 (8.0 – 16.1) | 0.11 |

[a]n=42

[b]n=44 (includes patients with HSN; two patients without renal involvement were excluded)

[c]Defined as urine protein to creatinine ratio > 20 mg/mmol or positive dipstick test (1+ to 3+)

**Table S6.** Common SNPs within the HLA region in genetic correlation analysis between HSP and IBD

| SNP | Gene | Gene description | Ensembl gene id | Chromosome | Start position | End position |
|---|---|---|---|---|---|---|
| rs115628514 | HLA-B | major histocompatibility complex, class I, B | ENSG00000234745 | 6 | 31269491 | 31357188 |
| rs4530903 | HLA-DRB1 | major histocompatibility complex, class II, DR beta 1 | ENSG00000196126 | 6 | 32578769 | 32589848 |
| rs9271344 | HLA-DRB1 | major histocompatibility complex, class II, DR beta 1 | ENSG00000196126 | 6 | 32578769 | 32589848 |
| rs35472814 | HLA-DQB1 | major histocompatibility complex, class II, DQ beta 1 | ENSG00000179344 | 6 | 32659467 | 32668383 |
| rs114169440 | HLA-DQB1 | major histocompatibility complex, class II, DQ beta 1 | ENSG00000179344 | 6 | 32659467 | 32668383 |
| rs35122881 | HLA-DQB1 | major histocompatibility complex, class II, DQ beta 1 | ENSG00000179344 | 6 | 32659467 | 32668383 |
| rs17427445 | HLA-DQB1 | major histocompatibility complex, class II, DQ beta 1 | ENSG00000179344 | 6 | 32659467 | 32668383 |
| rs34677551 | HLA-DQB1 | major histocompatibility complex, class II, DQ beta 1 | ENSG00000179344 | 6 | 32659467 | 32668383 |
| rs35900855 | HLA-DQB1 | major histocompatibility complex, class II, DQ beta 1 | ENSG00000179344 | 6 | 32659467 | 32668383 |
| rs115255544 | HLA-DQB1 | major histocompatibility complex, class II, DQ beta 1 | ENSG00000179344 | 6 | 32659467 | 32668383 |
| rs17212462 | HLA-DQB1 | major histocompatibility complex, class II, DQ beta 1 | ENSG00000179344 | 6 | 32659467 | 32668383 |
| rs17212483 | HLA-DQB1 | major histocompatibility complex, class II, DQ beta 1 | ENSG00000179344 | 6 | 32659467 | 32668383 |
| rs10484561 | HLA-DQB1 | major histocompatibility complex, class II, DQ beta 1 | ENSG00000179344 | 6 | 32659467 | 32668383 |
| rs17212553 | HLA-DQB1 | major histocompatibility complex, class II, DQ beta 1 | ENSG00000179344 | 6 | 32659467 | 32668383 |
| rs17427564 | HLA-DQB1 | major histocompatibility complex, class II, DQ beta 1 | ENSG00000179344 | 6 | 32659467 | 32668383 |
