# Supplementary material for: HLA-DQ and HLA-DRB1 alleles associated with Henoch-Schönlein purpura nephritis in Finnish pediatric population: a genome-wide association study
Source: Pediatr Nephrol. 2021 Feb 16;36(8):2311–8. doi: 10.1007/s00467-021-04955-7 (PMC8260528; doi:10.1007/s00467-021-04955-7)
Supplement: Supplementary file 4 — (PPTX 204 kb). [file 467_2021_4955_MOESM4_ESM.pptx]

## Slide 1
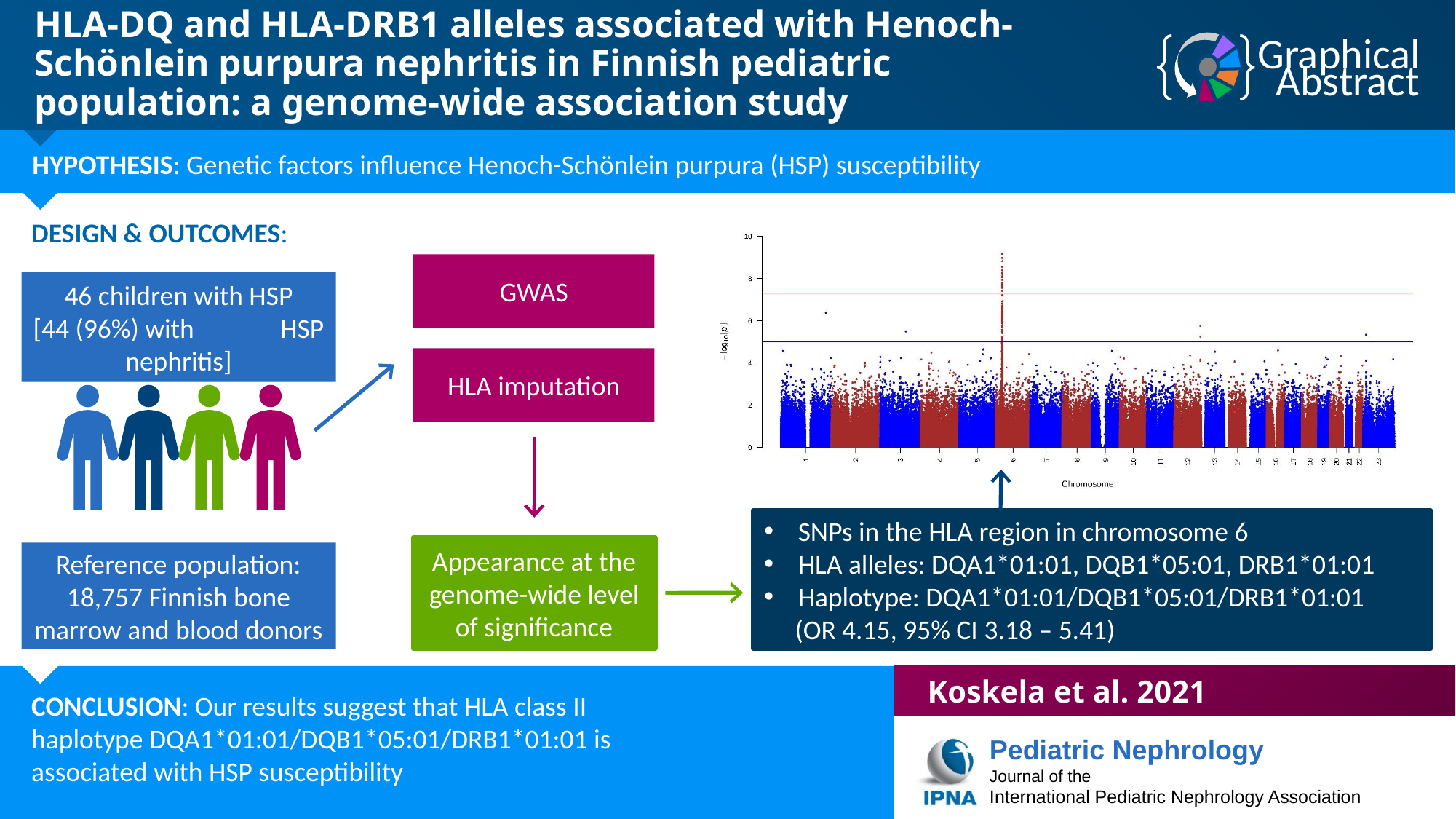

HLA-DQ and HLA-DRB1 alleles associated with Henoch-Schönlein purpura nephritis in Finnish pediatric population: a genome-wide association study
HYPOTHESIS: Genetic factors influence Henoch-Schönlein purpura (HSP) susceptibility
DESIGN & OUTCOMES:
GWAS
46 children with HSP
[44 (96%) with HSP nephritis]
HLA imputation
SNPs in the HLA region in chromosome 6
HLA alleles: DQA1*01:01, DQB1*05:01, DRB1*01:01
Haplotype: DQA1*01:01/DQB1*05:01/DRB1*01:01
 (OR 4.15, 95% CI 3.18 – 5.41)
Appearance at the genome-wide level of significance
Reference population: 18,757 Finnish bone marrow and blood donors
Koskela et al. 2021
CONCLUSION: Our results suggest that HLA class II haplotype DQA1*01:01/DQB1*05:01/DRB1*01:01 is associated with HSP susceptibility
